# How the COVID-19 Pandemic Impacted on Integrated Care Pathways for Lung Cancer: The Parallel Experience of a COVID-Spared and a COVID-Dedicated Center

**DOI:** 10.3389/fonc.2021.669786

**Published:** 2021-06-28

**Authors:** Giulia Pasello, Jessica Menis, Sara Pilotto, Stefano Frega, Lorenzo Belluomini, Federica Pezzuto, Anna Caliò, Matteo Sepulcri, Nunzia Luna Valentina Cernusco, Marco Schiavon, Maurizio Valentino Infante, Marco Damin, Claudio Micheletto, Paola Del Bianco, Riccardo Giovannetti, Laura Bonanno, Umberto Fantoni, Valentina Guarneri, Fiorella Calabrese, Federico Rea, Michele Milella, PierFranco Conte

**Affiliations:** ^1^ Medical Oncology Department, Istituto Oncologico Veneto IRCCS, Padova, Italy; ^2^ Department of Surgery, Oncology and Gastroenterology, University of Padova, Padova, Italy; ^3^ Section of Oncology, Department of Medicine, University of Verona School of Medicine and Verona University Hospital Trust, Verona, Italy; ^4^ University of Padova, Medical School, Department of Cardiac, Thoracic, Vascular Sciences and Public Health, Padova, Italy; ^5^ Department of Diagnostics and Public Health, University of Verona School of Medicine and Verona University Hospital Trust, Verona, Italy; ^6^ Radiation Oncology Department, Istituto Oncologico Veneto IRCCS, Padova, Italy; ^7^ Radiation Oncology Department, University of Verona School of Medicine and Verona University Hospital Trust, Verona, Italy; ^8^ Cardiovascular and Thoracic Department, University of Verona School of Medicine and Verona University Hospital Trust, Verona, Italy

**Keywords:** lung cancer, COVID-19, multidisciplinary team discussion, integrated care pathway, pandemic

## Abstract

**Introduction:**

The COVID-19 pandemic has proved to be a historic challenge for healthcare systems, particularly with regard to cancer patients. So far, very limited data have been presented on the impact on integrated care pathways (ICPs).

**Methods:**

We reviewed the ICPs of lung cancer patients who accessed the Veneto Institute of Oncology (IOV)/University Hospital of Padua (Center 1) and the University Hospital of Verona (Center 2) before and after the COVID-19 pandemic, through sixteen indicators chosen by the members of a multidisciplinary team (MDT).

**Results:**

Two window periods (March and April 2019 and 2020) were chosen for comparison. Endoscopic diagnostic procedures and major resections for early stage NSCLC patients increased at Center 1, where a priority pathway with dedicated personnel was established for cancer patients. A slight decrease was observed at Center 2 which became part of the COVID unit. Personnel shortage and different processing methods of tumor samples determined a slightly longer time for diagnostic pathway completion at both Centers. Personnel protection strategies led to a MDT reshape on a web basis and to a significant selection of cases to be discussed in both Centers. The optimization of patient access to healthcare units reduced first outpatient oncological visits, patient enrollment in clinical trials, and end-of-life cancer systemic treatments; finally, a higher proportion of hypofractionation was delivered as a radiotherapy approach for early stage and locally advanced NSCLC.

**Conclusions:**

Based on the experience of the two Centers, we identified the key steps in ICP that were impacted by the COVID-19 pandemic so as to proactively put in place a robust service provision of thoracic oncology.

## Introduction

A novel disease caused by the coronavirus, COVID-19, causing severe acute respiratory syndrome (SARS), named SARS-CoV-2, was classified as a pandemic by the World Health Organization (WHO) on 12 March 2020 (1). COVID-19 has proved to be a historic challenge for healthcare systems around the world (2, 3).

In particular, the management of cancer care soon became a crucial issue to which national and international oncology organizations replied with recommendations concerning patients receiving anticancer treatments. Recommendations included delaying active treatment administration based on a case-by-case risk/benefit evaluation, planning remote follow-up assessments, and limiting caregiver access to hospitals (4, 5).

Lung cancer is still the leading cause of cancer death worldwide, and it has been associated with a high risk of pulmonary complications and mortality linked to SARS-CoV2 infection (6).

Lung cancer patient care involves several health professionals and different units joined together in multidisciplinary teams. Integrated Care Pathways (ICPs) have been identified as an adequate tool for improving the management of lung cancer patient care (7–9). ICPs are currently widely used in hospitals for a structured and detailed planning of care, since they facilitate a systematic and continuous audit of clinical practices through quality indicators that investigate the three dimensions of quality: professional, organizational, and patient-oriented care (7–10).

Raising evidence on lung cancer care was published during the COVID-19 pandemic, but, to the best of our knowledge, very limited data were presented on the impact on ICP. The ICP for lung cancer has been active in the Veneto region since 2017 and measures these patients’ quality of care (11).

We joined the experience of two Centers in the Veneto region, one of the regions in Italy that was most hit by the COVID-19 pandemic, with the primary aim of investigating the impact of COVID-19 on the regional ICP for lung cancer patients.

## Materials and Methods

The study was designed on 30 April 2020 with the primary aim of investigating the difference in terms of diagnostic and therapeutic procedure volumes and timings in a two-month observation period: 1 March–30 April 2019, and 1 March–30 April 2020.

This time frame has been selected in order to catch the different impact of the pandemic on two different centers, at the beginning of the infection curve rise in Italy; indeed, the pandemic curve reached a peak between March and April 2020, while a significant decrease was observed at the beginning of May.

The working group comprised all the physicians and healthcare researchers involved in the management of lung cancer patients in the two Veneto Region Centers: medical oncologists, pulmonologists, thoracic surgeons, pathologists, and radiation oncologists from the Veneto Institute of Oncology (IOV) and the University Hospital of Padua (named Center 1) and the University Hospital of Verona (named Center 2). While Center 1 one was only partially dedicated to COVID-19 patient care, Center 2 became a COVID-dedicated hospital on 13 March 2020 (12).

Indicators were identified by using group facilitation techniques designed to explore the level of consensus among a group of experts and to aggregate judgments into refined agreed opinions. Facilitation includes a set of functions or activities carried out before, during, and after a meeting to help the group achieve its own outcomes. Facilitative functions may be accomplished by group members or leaders or by an external facilitation specialist. This method is usually used during the meetings by the ICP working groups in order to assess among all specialists which indicators are best suitable for different purposes.

A total of sixteen indicators (**Supplementary Table 1**) were selected taking into account their availability, reproducibility, significance, and measurability. These indicators were agreed upon and also chosen based on the Veneto ICP (11), literature evidence (7–10), international guidelines (13–15), and the expert opinion of the hospital’s multidisciplinary lung cancer team. The deadline for data collection was set for 22 May 2020.

We retrospectively reviewed the ICPs of consecutive lung cancer patients who were referred to the two Centers during the two-month observation period. The last follow-up was 30 April 2020.

The clinical features of referred patients were not this study’s primary interest. Therefore, all data were collected in an anonymous form.

Data on activity volumes from oncology, pulmonology, radiation therapy, and thoracic surgery were derived from electronic medical records, institutional electronic tracking systems usually employed for administrative purposes, and multidisciplinary team registries. Data on patients’ enrollment in clinical trials were gathered through our electronic database by data managers fully dedicated to lung cancer clinical trials. Data of activity volumes of the Center 1 pathology unit were extracted through Armonia Software (Dedalus Healthcare Systems Group) version 15.0.7.1, while those of the Center 2 pathology unit were gathered through Pathos Software. All data were further confirmed by manually examining daily activity lists.

Indicators to assess each Center’s activity volumes were reported as total numbers, percentages, or ratios; indicators to assess different performance times were calculated as median and mean with working day ranges. The total number of patients evaluated for each indicator (*i.e.* the denominator) varied depending on the indicator’s content.

**Figure 1:**
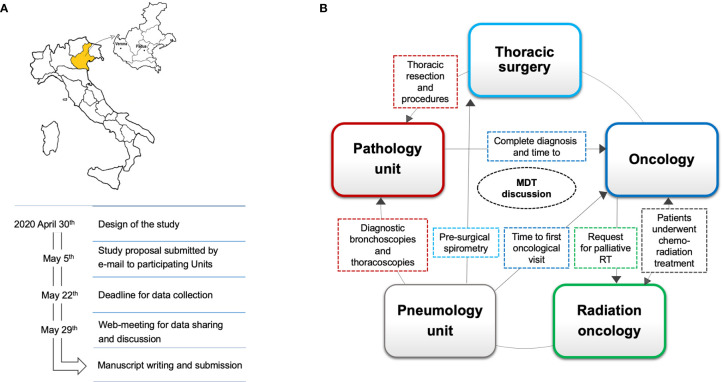
Study design and data collection involving two Centers (Center 1 in Padua; Center 2 in Verona) in the Veneto region, marked here in yellow. The study’s main steps were pursued *via* telematics, from the study design to data collection and sharing results, in compliance with pandemic containment measures **(A)**. The impact of COVID-19 on lung cancer patient care was assessed through indicators (dashed lines), identified and selected from regional Integrated Care Pathways (ICPs) and from an expert panel on the basis of relevance to healthcare providers’ workloads. The direction of the arrows depicts how these cancer specialists interact with each other in the context of ICPs, while the color of the indicator is the same as that of the healthcare provider whose activity is influenced by **(B)**. MDT, multidisciplinary team.

A web meeting for data sharing and discussion took place on 29 May 2020 before manuscript writing and submission. The study design and timeline are summarized in **Figure 1**.

The performance time in 2019 and 2020 was calculated through median values and compared by means of the Mann–Whitney Rank Sum Test. Activity volumes were compared through a two-tailed binomial test used to determine whether the probability of each indicator is equal to 1/2 in the two periods and a Pearson’s Chi-squared test with simulated p-value to compare two observed proportions. Specifically, when comparing the 2019 and 2020 activity volumes we supposed that, of the total number of procedures (y1 + y2), a portion y1 was performed in the first year and a portion y2 in the second year.

## Results

The Veneto Institute of Oncology, which includes medical and radiation oncology, was not defined as a COVID-19 hospital during the entire pandemic; the thoracic surgery, pneumology, and pathology units of the University Hospital of Padua were partially involved in the diagnosis and care of COVID-19 patients, even though some personnel were specifically dedicated to endoscopic diagnostic procedures for cancer patients.

On the other hand, the Verona University Hospital Trust was converted into a COVID hospital during the pandemic’s peak. The thoracic surgery, pneumology, and oncology units were heavily involved in COVID-19 patient care.

### Activity Volumes of Pneumology Units and Timing for NSCLC Patients

The absolute number of diagnostic bronchoscopies performed at the Center 1 pneumology unit was 29 in 2019, increasing to 43 in 2020 (*p* value = 0.1249), while those performed at Center 2 decreased from 16 in 2019 to 8 in 2020 (*p* value = 0.1516).

**Figure 2:**
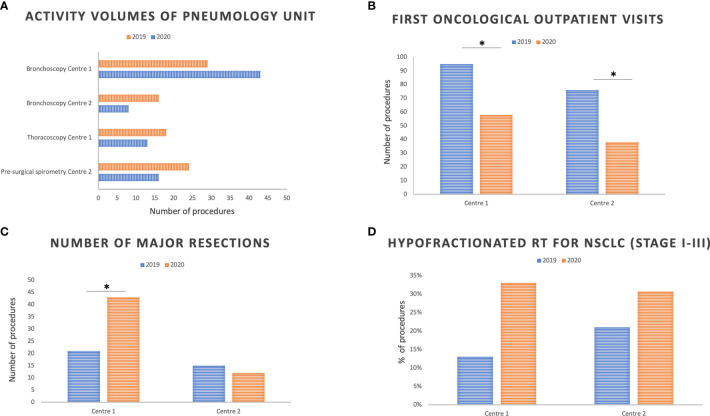
Pneumology **(A)**, medical oncology **(B)**, thoracic surgery **(C)**, and radiation oncology **(D)** activity volumes at the two Centers in March and April 2019 compared with March and April 2020. RT, radiotherapy. *Indicates statistically significant differences between 2019 and 2020; *p* significance level: 0.05.

The second indicator was differentiated between the two Centers in order to have a reliable assessment of the activities in the two time periods. The absolute number of thoracoscopies showed a slight non-significant decrease in Center 1, with 18 procedures in 2019 and 13 in 2020 (*p* value = 0.4731). Similarly, pre-surgical spirometries in Center 2 numbered 24 in 2019, decreasing to 16 in 2020 (*p* = 0.2682) (**Figure 2A**).

**Figure 3:**
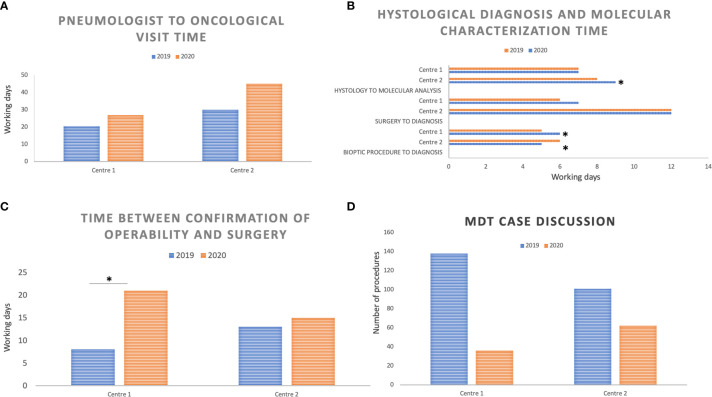
Median performance time reported in working days and compared between March and April 2019 *versus* March and April 2020 at the two Centers: **(A)** Time interval between the pneumology assessment and the first oncological visit; **(B)** Time interval between the biopsy or surgical procedure and the histological diagnosis, and between the histological diagnosis and molecular results; **(C)** Time interval between the confirmation of operability based on functional assessment and surgery (lobectomy or pneumonectomy); **(D)** Number of cases discussed by the multidisciplinary team (MDT) at the two Centers in March and April 2019 compared with March and April 2020. *Indicates statistically significant differences between 2019 and 2020; *p* significance level: 0.05.

Finally, the median time between the first visit to the pneumologist and the first oncological visit in Center 1 was 20.5 days in 2019 and 27 in 2020 (*p* value = 0.160), while this was 30 days in 2019 and 45 days in 2020 in Center 2 (*p* value < 0.0001) (**Figure 3A**). The benchmark established by the Regional ICP for this indicator is 28 working days.

### Histological Diagnosis and Molecular Characterization Timing

The Center 1 pathology unit observed a median time from biopsy procedure to histological diagnosis of 5 working days (wd) in 2019 and 6 wd in 2020 (*p* value < 0.001); in Center 2, the median time from biopsy to histological diagnosis was 6 days in 2019 and 5 days in 2020 (*p* value = 0.014). In both sites, a significant reduction in diagnoses from biopsy samples was observed. Indeed, in Center 1, histological samples from tumor biopsies totalled 215 in 2019 and 171 in 2020 (*p* value = 0.0285), with an overall 20% reduction; in Center 2, biopsy samples decreased from 79 in 2019 to 37 in 2020 (*p* value = 0.0001), with an overall 53% reduction.

Conversely, the diagnostic process from surgical specimens did not change significantly in the two Centers in 2020 compared to 2019, both in terms of the number of surgical specimens analyzed and in terms of the median time from surgical procedure to histological diagnosis. In both Centers, the median time for a histological report was within the 20 days set as a benchmark by the Regional ICP: 6 wd in 2019 and 7 wd in 2020 at Center 1 (*p* = 0.058), 12 wd in both periods at Center 2 (*p* value = 0.94).

In the same way, molecular analyses (ROS1, ALK, and PD-L1 immunohistochemistry; ALK and ROS1 FISH, EGFR mutational analysis through real-time PCR) overlapped numerically in 2019 and 2020 in both Centers; the median time between histological diagnosis and molecular characterization results was only slightly longer in Center 2 (from 8 to 9 days, *p* value = 0.02) while in Center 1, this was 7 days in both periods (*p* value = 0.440) (Regional ICP benchmark: 10 wd) (**Figure 3B**).

### Oncological Activity Volumes and the Therapeutic Pathway of NSCLC Cancer Patients

We analyzed the absolute number of first oncological outpatient visits at the two Centers, and we observed a significant reduction in 2020 compared to 2019. Indeed, first visits in Center 1 amounted to 95 in 2019 and 58 in 2020 (*p* value = 0.0035), a 39% reduction; in Center 2, 76 first visits were held in 2019 and 38 in 2020, with a 50% reduction (*p* valu*e* = 0.0005) (**Figure 2B**).

In the same way, the number of patients enrolled in clinical trials, calculated as the ratio between the number of patients enrolled in clinical trials and the number of active clinical trials, decreased in 2020. Forty-nine patients were enrolled in 13 active trials at Center 1 in 2019 (ratio 3.8), while 34 patients were enrolled in 14 active clinical trials in 2020 (ratio 2.4). Center 2 enrolled 10 patients in 10 clinical studies in 2019 (ratio 1), and eight patients in 10 clinical trials in 2020 (ratio 0.8).

Access prioritization, soon established for both Centers, led to a reduction in the proportion of patients who received systemic anticancer treatment 30 days before death, calculated as the ratio between the number of patients who died within 30 days from the last systemic treatment administration and the number of patients undergoing active systemic treatment. From among 296 active treatments ongoing at Center 1 in 2019 and 335 in 2020, 12 patients (4%) died within 30 days from the last treatment administration in 2019 and eight (2%) in 2020 (*p* value = 0.2684). In Center 2, seven patients (3%) out of 222 in 2019 and four (2%) out of 213 in 2020 died within 30 days from the last treatment administration (*p* value = 0.5517) (Regional ICP benchmark: lower than 10%).

### Activity Volumes, Timings and Mortality Rates From Thoracic Surgical Procedures

As far as timing and activity volumes of thoracic surgery units are concerned, we first assessed an indicator from the ICPs of the Veneto region, namely the median time between the confirmation of operability based on functional assessment and surgery (lobectomy or pneumonectomy).

While the median time in Center 1 was prolonged from 8 working days in 2019 to 21 working days in 2020 (*p* value = 0.009), times overlapped in Center 2 (13 working days in 2019, range 2–35; 15 working days in 2020, range 4–43; *p* value = 0.557) (**Figure 3C**).

Moreover, thoracic surgery in Center 1 saw an increase in the percentage of major resections, calculated as the number of NSCLC major resections out the total number of procedures: from 21 in 2019 to 43 in 2020 (*p* value = 0.0081). Conversely, a 20% reduction in major resections was observed in Center 2: from 15 in 2019 to 12 in 2020 (*p* value = 0.7011) (**Figure 2C**). No difference in mortality rates, calculated within 30 days from major anatomical resection, was observed in the two Centers between 2019 and 2020 (0%) (Regional ICP benchmark: lower than 10%).

### Radiation Therapy in Locally Advanced and Palliative Locoregional Treatments in Metastatic NSCLC

Radiation oncologists carefully assessed three performance indicators which were potentially impacted by the COVID-19 pandemic. No significant difference in terms of percentage of concomitant chemo-radiotherapy (cCTRT) in stage III NSCLC patients was observed. This was calculated as the ratio between the concomitant chemo-radiotherapy treatments and the overall chemo-radiotherapy treatments (concomitant *plus* sequential: cCTRT). Center 1 observed seven cCTRT (78%) out of nine cCTRT in 2019, and six (86%) out of seven in 2020 (*p* value = 1), while Center 2 experienced four (50%) out of eight cCTRT in 2019 and three (60%) out of five in 2020 (*p* value = 1). Both Centers increased the percentage of hypofractionated regimens on overall treatment plans: from 13 (3/23) to 33% (8/24) in Center 1 between 2019 and 2020 (*p* value = 0.1684), and from 21 (10/48) to 31% (10/32) in Center 2 (*p* value = 0.4233) in the same time periods (**Figure 2D**).

Finally, a reduction in palliative treatments (namely, disease relief symptoms on the brain, bone, and chest) was observed in both Centers during the COVID peak period. While palliative treatments in 2019 totalled 54 in Center 1 and 21 in Center 2, these dropped to 44 (*p* value = 0.3634) and 17 (*p* value = 0.6271) in 2020, respectively, in the two Centers.

### Multidisciplinary Team Discussion

Across March and April 2019, eight MDT meetings took place in Center 1 (in an 8 week period) wherein 138 patients were discussed. In March–April 2020, four meetings took place (one physical and three web-based) in a 9 week period, during which 36 patients were discussed (p < 0.0001). The COVID-19 pandemic determined a 74% reduction in the number of patients discussed. Similarly, in Center 2, eight MDT meetings took place in 2019 (in an 8 week period) wherein 101 patients were discussed, while seven MDT meetings took place in 2020, during which 62 patients were discussed (p = 0.0028), with a final 39% reduction in the number of patients discussed (**Figure 3D**).

## Discussion

By the end of April 2020, Italy had already been badly hit by COVID-19, with 201,505 overall confirmed cases and 27,359 deaths since the beginning of this pandemic (source: National Health System data) (15). Cancer patients are more susceptible to this infection compared to healthy people and non-cancer patients due to the systemic malignancy-related immunosuppressive state and to active disease-oriented treatments, such as chemotherapy or immunotherapy, radiotherapy and surgery (16–20). Therefore, cancer care professionals were called upon to cautiously manage this emergency in the everyday balance between risks and benefits in treatment planning (21, 22). Nevertheless, such uncertainty caused patients to feel abandoned, worsened disease-related distress, and led to patients leaving life-saving treatments, as recently reported in 15–20% of cases (23).

To build and share knowledge, several real-world data was collected with the aim of counting the number of infected patients, hospital and Intensive Care Unit admissions, and to measure the mortality and acquirement of immunity (24, 25).

However, to the best of our knowledge, no focused effort has been made to date to retrospectively measure the impact on local cancer management and to assess possible shortages of non-COVID-19-related healthcare provisions (26). Moreover, the majority of reports focused on single-center experiences (27, 28).

The present work focuses on the integrated care pathways of NSCLC management. The aim is to report data on multidisciplinary performance volumes and timing of NSCLC patients referred to two Italian Centers, one COVID-spared and one COVID-dedicated, in March and April 2020 and to compare the data with the same 2019 period.

Even though both pneumology units had to reorganize their activities to cope with the management of COVID-19 patients, a different impact of the pandemic on performance volumes and timings was observed between the two Centers. The Center 1 pneumology unit had a slight decrease in the number of thoracoscopy procedures requiring an operating theater. However, since a priority pathway for cancer patients with dedicated specialized personnel for endoscopic diagnostic procedures was established, the pure number of procedures actually increased in 2020 compared to 2019. On the other hand, the Center 2 unit was entirely involved in COVID-positive patient diagnoses and care and, subsequently, had a higher volume of bronchoscopy and spirometry procedures.

For both Centers, the time between the first pulmonologist and the first oncological visits was slightly prolonged due to the longer time needed for staging and diagnostic pathway completion. Similar to the experience of pathological departments in other Italian Centres (29), the activities of both pathology departments were affected since technical and administrative personnel were reduced on-site and moved to smart working, thus limiting the workforce available for sample processing. Pathologists and technicians were also involved in the *post-mortem* examination of suspected or certain COVID-19-positive patients, which was time-consuming and personnel-intensive. Moreover, cytological samples from bronchoalveolar lavage (BAL) were first processed through alcohol and formalin treatment and only analyzed later in order to avoid potential exposure to the virus (30–32).

A significant reduction in first oncological evaluations was observed at both Centers, and this was mostly due to a decrease in visits from outside the region and the optimization of patient access to the oncological unit, which led medical oncologists to postpone clinical evaluation when all diagnostics and staging was complete. In accordance with the recommendations of the main national and European guidelines (4, 5), a careful evaluation of treatment administration was performed for frail patients on the basis of age and comorbidities, and also for further lines of treatment, with particular reference to those regimens lacking strong evidence of survival benefits. As a consequence, we observed a reduction in patient deaths within 30 days from the last anticancer treatment administration, which was established as a relevant indicator by Regional ICPs.

Once again, the different trend in major resection numbers between the two Centers was a consequence of the two units’ different levels of involvement in COVID-19-positive patient care. Indeed, while the availability of operating rooms increased in Center 1, where priority was given to cancer patients suitable for major interventions, operating rooms were temporarily closed to cancer patients and subsequently opened with a limited flow in Center 2. The longer time observed in Center 1 between the operability indication and surgical resection was mainly due to the minimization of patient access to preoperative tests and anesthesiological assessment.

Departmental reorganization and limiting patient access to the hospital also involved the radiation therapy unit, leading to an increase in hypofractionated regimens (33). This was in line with the Italian experience recently reported by the Italian Association of Radiotherapy and Clinical Oncology (AIRO), where hypofractionation was considered as one of the basic strategies to efficiently cope with the disruption caused by the pandemic (34).

Additionally, some patients showed reduced compliance to repeated access to hospital for locoregional treatments with palliative intent, thus leading to the optimization of best supportive care at home.

Overall, the indicators established by the Regional ICP and selected for the purpose of this study confirmed an adequate management and diagnostic–therapeutic pathway for cancer patients; indeed, from the six of them, only one indicator exceeded the recommended benchmark, and this involved the Center 2 pneumology unit which was converted into a COVID-dedicated unit.

MDT meetings at Center 1 were more impacted than Center 2, even because the earlier spread of COVID-19 was focused in Padua; moreover, in Center 2, MDT was promptly switched to the web-based format thus allowing a higher number of web meetings to take place. The decrease observed in the number of discussion cases was overall in line with the reduction in oncological visits and diagnostic procedures during the pandemic’s peak and to a better selection of cases to be discussed where more than two specialists were needed.

In order to reduce patient access to healthcare units and to select patients for locoregional and systemic treatments, multidisciplinary team discussion should be maintained as a basic requirement for good clinical practice in lung cancer patient management, potentially impacting patients’ survival (35, 36).

The optimization of telemedicine and resource allocation for easy-to-use software would allow patients’ clinical documents and iconography to be transferred between physicians and between physicians and patients. These are starting points for innovative healthcare practice and would also help to reassure patients on the presence and reliability of oncologists.

Worldwide teamwork in the sharing of clinical and autoptic data on lung cancer patients affected by COVID-19 and a longer follow-up of such patients may help clinicians to efficiently shape their practice for continuous care in oncology and the protection of frail subpopulations (26).

Based on the experience of the two Centers, we identified the key determinants for a robust provision of thoracic procedures: pre-defined plans for epidemic response; aggressive early action to “flatten the curve”; the ability to separate resources between the management of COVID-19 (or any epidemic) and routine clinical services; prioritization of thoracic surgery; and, the volume of COVID-19 cases in that region.

Although the impact of health reorganization measures on patients’ survival is not the aim of the present study, this issue is currently under investigation at national level where lung cancer patients’ outcome has been investigated with longer follow-up.

The limitations of this analysis are certainly related to the study’s retrospective nature and the limited number of Centers involved; nevertheless, in our opinion, this analysis presents a good picture of the experience acquired by the two Centers in one of the Italian regions worst-hit by the COVID-19 pandemic.

Some approaches for facing the impact of pandemic on health system, which were improved only in subsequent months of 2020 and finally became routine strategies during the second peak of 2021, include a prompt plan for protection and monitoring of personnel, who should also be divided into different paths according to access to COVID or no-COVID departments; production of time and personnel-sparing procedures for patients’ sample management, which take under consideration the central role of the pathologist and pulmonologist both in COVID infection and cancer diagnostic process; dynamic update of guidelines on systemic and locoregional treatment of lung cancer patients in order to warrant early start of the diagnostic–therapeutic pathway and to avoid hospital access not finalized to survival improvement; finally, patients’ empowerment to early cancer symptoms and signs referral and diagnostic and staging procedure compliance in order to limit advanced stages at the clinical presentation.

To conclude, the lessons learned will improve the ICP through more flexibility and awareness of pivotal points and will go a long way to ensure continuity of care by caregivers to cancer patients.

## Data Availability Statement

The raw data supporting the conclusions of this article will be made available by the authors without undue reservation.

## Ethics Statement

Ethical review and approval was not required for the study on human participants in accordance with the local legislation and institutional requirements. Written informed consent for participation was not required for this study in accordance with the national legislation and the institutional requirements.

## Author Contributions

All authors listed have made a substantial, direct, and intellectual contribution to the work and approved it for publication.

## Conflict of Interest

The authors declare that the research was conducted in the absence of any commercial or financial relationships that could be construed as a potential conflict of interest.
