# Supplementary material for: How the COVID-19 Pandemic Impacted on Integrated Care Pathways for Lung Cancer: The Parallel Experience of a COVID-Spared and a COVID-Dedicated Center
Source: Front Oncol. 2021 Jun 28;11:669786. doi: 10.3389/fonc.2021.669786 (PMC8273534; doi:10.3389/fonc.2021.669786)
Supplement: Supplementary file 1 [file Table_1.docx]

**Table 1. Integrated Care Pathways indicators**

| **Unit** | **Indicator** |
| --- | --- |
| **Pneumology** | - Absolute number of diagnostic bronchoscopies - Absolute number of medical thoracoscopies (Padua only) - Absolute number of pre-operative spirometries (Verona only) - Time between the first visit of the pneumologist and the first oncological visit* |
| **Pathology** | - Time between the diagnostic procedure and diagnosis - Time between the surgery and diagnosis* - Time between the histological diagnosis and the molecular characterization* |
| **Surgery** | - Time between confirmation of operability based on functional assessment and surgery (lobectomy or pneumonectomy)* - Percentage of major resection for NSCLC - Mortality rate within 30 days from surgery in major anatomical resection * |
| **Radiation oncology** | - Percentage of radiation treatments concomitant to chemotherapy (cCTRT) for stage III NSCLC patients: number of cCTRT/total number of radiation treatments concomitant or sequential to chemotherapy - Number of radiation treatments for NSCLC (stage I-III) with hypofractionation compared with standard regimen - Absolute number of palliative locoregional treatments |
| **Medical oncology** | - Absolute number of first outpatient visits - Patients enrollment in clinical trials - Percentage of patients who received systemic treatment in the 30 days before death*^#^ |
| **Multidisciplinary team** | - Absolute number of patients discussed |

** ICP Veneto region*

^#^ *observation period 1^st^ March to 15^th^ April*
